# The Role of Estrogen Signaling and Exercise in Drug Abuse: A Review

**DOI:** 10.3390/clinpract14010012

**Published:** 2024-01-08

**Authors:** Rania Ahmed, Samuel Zyla, Nikki Hammond, Kenneth Blum, Panayotis K. Thanos

**Affiliations:** 1 Department of Psychology, University at Buffalo, Buffalo, NY 14203, USA; raniaahm@buffalo.edu; 2 Behavioral Neuropharmacology and Neuroimaging Laboratory on Addictions (BNNLA), Research Institute on Addictions, Department of Pharmacology and Toxicology, Jacobs School of Medicine and Biomedical Sciences, Buffalo, NY 14203, USA; samuelzy@buffalo.edu (S.Z.); nikkiham@buffalo.edu (N.H.); 3 Division of Addiction Research Education, Center for Sports, Exercise and Mental Health, Western University Health Sciences, Pomona, CA 91766, USA; drd2gene@gmail.com

**Keywords:** estrogen, exercise, drug abuse, sex differences, ovariectomy

## Abstract

Background: Discovering how sex differences impact the efficacy of exercise regimens used for treating drug addiction is becoming increasingly important. Estrogen is a hormone believed to explain a large portion of sex differences observed during drug addiction, and why certain exercise regimens are not equally effective between sexes in treatment. Addiction is currently a global hindrance to millions, many of whom are suffering under the influence of their brain’s intrinsic reward system coupled with external environmental factors. Substance abuse disorders in the U.S. alone cost billions of dollars annually. Review Summary: Studies involving the manipulation of estrogen levels in female rodents, primarily via ovariectomy, highlight its impact regarding drug addiction. More specifically, female rodents with higher estrogen levels during the estrus phase increase cocaine consumption, whereas those in the non-estrus phase (low estrogen levels) decrease cocaine consumption. If estrogen is reintroduced, self-administration increases once again. Exercise has been proven to decrease relapse tendency, but its effect on estrogen levels is not fully understood. Conclusions: Such findings and results discussed in this review suggest that estrogen influences the susceptibility of females to relapse. Therefore, to improve drug-abuse-related treatment, exercise regimens for females should be generated based on key sex differences with respect to males.

## 1. Introduction

The investigation of sex differences regarding exercise and drug addiction has gained more popularity as exercise strategies specific to sex contribute to a more comprehensive understanding of drug addiction and inform the development of targeted treatments. There is a high burden of drug abuse, with the U.S. Substance Abuse and Mental Health Services Administration (SAMHSA) reporting that 6.5% of the population met the DSM-5 criteria for having a substance use disorder in 2021. Many do not receive treatment [1]. Sex hormones have been shown to play a role in synaptic plasticity, neurogenesis, and neurotransmitter systems—the underlying mechanisms of drug addiction [2]. Estrogen is one sex hormone believed to be responsible for observed sex disparities seen in drug addiction. It may influence exercise regimens aiming to serve as therapeutic interventions in treating drug addiction by modulating the mesolimbic dopamine pathway [2]. This narrative review will investigate the sex differences pertaining to drug abuse, as well as the observed effects of estrogen removal and treatment, and how this correlates with exercise regimens. Of course, treatment for drug addiction is multifaceted and involves a combination of behavioral therapy, medication, and ongoing support. Exercise is one part of this process that, in combination with other methods, can aid in supporting an individual through recovery, although clinical research on this area is lacking. The role of exercise—in terms of providing a dependable option for the treatment of drug addiction—greatly relies on the fine-tuning of these findings in relation to estrogen levels and its other related physiological implications to better understand the potential role sex-specific variables may have in responses to exercise and drugs [2]. The focus of this paper is on estrogen; however, we acknowledge that there are other hormones and neurotransmitters involved in modulating the effects of exercise and drug abuse. In this narrative review, we conducted a comprehensive search utilizing various search engines, including PubMed and the University at Buffalo’s Library database. This search yielded 90 relevant results. All published articles were peer-reviewed and published, dating from 1997 to the present, featuring robust statistical analyses.

## 2. Drugs of Abuse: Mechanisms of Action

### 2.1. General Mechanisms

The HPA axis of the neuroendocrine system is crucial in neurobehavioral and psychobiological processes of drug addiction [3]. Increasing evidence has pinpointed the mesolimbic DA circuit within this pathway as mediating addiction to certain drugs [4]. Since most drugs of abuse activate the DA system and increase DA brain transmission, regulation of this system could help treat drug addiction. It is important to note that drug addiction due to repeated exposure is different from occasional drug use, and chronic drug use can induce neuroadaptations involving several neural systems [5]. Research has shown that the glutamatergic system in the NAc and PFC plays an important role in mediating drug seeking and relapse following a period of abstinence [6]. Negative reinforcing effects associated with abstinence such as withdrawal and negative affect also contribute to drug addiction via the anti-reward/stress system in the extended amygdala [7,8]. They trigger compulsive drug use, drug-seeking behavior, and relapse [9]. Individuals suffering with substance abuse disorder often experience comorbid mental disorders; therefore, the emotional disturbances of withdrawal can worsen pre-existing mental disorders, which, in turn, may contribute further to a cycle of increased drug use [10]. A literature search by Ahmed et al. (2023) examines the vulnerabilities that exist within psychoactive substance use, including heritable alterations of epigenetic biomarkers in both clinical and preclinical models [11]. Furthermore, Koob and Volkow (2016) provide an in-depth review of addiction in brain neurocircuitry by exploring neurobiological mechanisms of motivation, executive function, and negative affect [12]. 

### 2.2. Alcohol

Maladaptive patterns of alcohol consumption are typically associated with increased activation of the amygdala and decreased activation of the medial prefrontal cortex (mPFC). Alcohol stimulates GABA_A_ receptors, which plays a role in cognitive deficits associated with increased alcohol intake and dependency. On the other hand, alcohol stimulates NMDA receptors, which alters neuroplasticity and contributes to cravings during withdrawal [13]. The medial preoptic area of the brain regulates the expression of gonadal hormones such as estrogen, which increases GABA_A_ expression in the region [14]. The endocannabinoid (ECB) system is an important component in the stress response and is tightly regulated by glucocorticoids and corticotropin-releasing factor (CRF) [15]. Deficits of ECB signaling in the basolateral amygdala (BLA) and ventral medial prefrontal cortex (vmPFC) may contribute to neuroadaptations, leading to withdrawal-induced anxiety in males, and ovariectomy (OVX) may cause similar disruptions in females that can be a driving force for relapse and drug-seeking behaviors. Additionally, OVX has been shown to elicit alcohol-withdrawal-induced disruptions in the ECB system in female rats that mirror those observed in male rats, without altering anxiety-like behavior [16]. Specific data indicate that sex hormones like estrogens protect against changes in corticolimbic ECB signaling but do not cause a hindrance to the anxiety experienced during the withdrawal stage. Thus, mechanisms accounting for alcohol-withdrawal-induced anxiety are most likely attributed differently to each sex [16].

### 2.3. Cocaine

Elevated cocaine use leads to enhanced signaling through dopamine D1 and D2 receptors which is responsible for the activation of intracellular signaling pathways coupled with G proteins. In this signaling cascade, G proteins activate or inhibit cyclic AMP-dependent protein kinase (PKA) [17]. This subsequently triggers the generation of cFos, which is an immediate early gene responsible for encoding a transcription factor believed to facilitate enduring alterations in neural function [17]. These immediate early genes trigger a number of short-term neuroplastic changes that, together, play a role in increased prefrontal cortex (PFC) excitability and neurotransmission, which contribute to the acute cognitive effects of cocaine [17]. In rats experiencing cocaine withdrawal, impairment of the reward system in the brain has been observed. This has been likened to depressive withdrawal symptoms observed in humans [18]. These symptoms are accompanied by increased levels of CRF in the limbic system. Similarly, CRF antagonists have been demonstrated to effectively alleviate adverse symptoms of withdrawal. Withdrawal symptoms are more pronounced in female cocaine abusers, which may contribute to an increased tendency for females to relapse [18]. An increased vulnerability to cocaine abuse can be explained by the sex differences in enhanced dopamine release shown in the rat estrous cycle, with females forming stronger and longer-lasting associations to drug cues [19]. 

### 2.4. Cannabinoids

Cannabinoid receptor 1 (CB1) and its endogenous ligands are expressed throughout the mesocorticolimbic pathway, and in the brain regions involved in decision-making, withdrawal symptoms, and relapse [20,21]. Within the mesocorticolimbic pathway, the ventral tegmental area (VTA) projects via DA neurons to the nucleus accumbens (NAc) and the forebrain. CB1 receptors in the VTA modulate DA release elicited by cannabinoid drugs. Increases in DA are seen after other prototypical drugs of abuse, such as morphine, nicotine, and ethanol [21]. Females do not respond to cannabinoids in the same way as males and show greater withdrawal symptoms. Sex differences in cannabinoid effects may be explained by ovarian hormones and interactions between the ECB system and hypothalamic–pituitary–adrenal (HPA) axis [2]. Intact female rats have reduced levels of CB1 in the hypothalamus and PFC. OVX females and intact males have higher levels of CB1 in the amygdala [2]. Additionally, the ECB system interacts with the hypothalamic–pituitary–ovarian (HPO) axis such that cannabinoids can suppress gonadal function, which can inhibit estrogen release [22]. Opioids also play a similar inhibitory role in the HPO axis, leading to lower levels of sex hormones [23].

### 2.5. Heroin/Opioids

Antinociception is critically modulated by opioid systems, as opioid peptides and receptors are found throughout nociceptive neural networks and in areas of the central nervous system associated with reward and emotion [24]. There are four different opioid receptor systems: mu, delta, kappa, and opioid-receptor-like 1 (ORL1) [24]. All four opioid receptors have been associated with behavioral effects, including analgesia, reward, depression, anxiety, and addiction. Tolerance to acute effects of the drug develops in response to prolonged opioid use, which can result in physical and psychological dependence [24].

## 3. Sex Differences in Drug Abuse

### 3.1. Human Studies 

Predisposition to drug dependence is a complex, multifaceted topic that involves biopsychosocial factors. Many of these factors may contribute to gender differences seen in substance abuse and addiction. Societal expectations shape gender roles and norms, which can influence accessibility to and experiences with substances. Additionally, the prevalence and nature of stress, mental health issues, and co-occurring diseases manifest differently in men and women, often leading to varying methods to cope and manage them. Recognizing these sex differences has important implications for determining the clinical effectiveness of targeted treatments and interventions. Research has shown that women experience greater adverse drug reactions compared to men. Women have longer periods of abuse after abstinence and are more likely to relapse due to depression and negative effects [25]. Studies have found that women have a higher rate of escalation compared to males, which allows them to stabilize at a higher dose, and are more likely to relapse [26]. Hormonal differences between men and women may play a role in drug dependence. Evidence from human studies reflect that the ovarian hormones, estrogen and progesterone, modulate vulnerability for females [27], and that estradiol may be required for features of psychostimulant addiction [28,29]. Estrogen signaling pathways interact with drug pharmacodynamic and/or pharmacokinetic pathways, contributing to adverse drug reactions in women [30]. Generally, metabolism and body composition differ between men and women, with women having a higher body fat percentage and lower lean muscle mass, which impacts absorption, distribution, and metabolism of a drug. Similarly, variations in enzyme activity impact the breakdown of a drug by the body. For example, hepatic cytochrome P450 enzymes are more numerous in women than in men. This variation can affect the breakdown of drugs in the body, thereby resulting in differences in drug side effects and therapeutic effects [30]. 

Hormonal fluctuations throughout the menstrual cycle are also responsible for mood changes and may be related to changes in motivation and addiction behavior [31]. Substance use is utilized to reduce the negative affect that is heightened at certain points during the cycle and/or enhance positive affect. A systematic review by Joyce et al. delves into the relations between menstrual cycle phase and female addictive behaviors [31]. Current research looking at the influence of menstrual cycle phase on the effects of alcohol is inconsistent, and findings are often conflicting, often due to differing methodologies, and many factors need to be considered such as hormonal contraceptives, Premenstrual Syndrome, and history of alcohol use (Table 1). Refer to Terner et al. (2006) and Warren et al. (2021) for a further analysis [32,33]. Few studies with opioid treatments have resulted in inconsistent findings, with some finding no cycle differences. Further research has found a significantly increased sensitivity to adverse effects during the luteal phase as compared to the follicular phase and men, as well as increases in cortisol and prolactin [32,34]. Consistent findings have been observed in relation to stimulant drugs such as cocaine, such that mood-altering effects are significantly observed in the follicular phase compared to the luteal [32,35]. Much research using nicotine has found effects on intake and behavior/mood, although inconsistencies remain. Elevated negative affect and behavior, as well as a greater tendency to relapse, have been observed in the follicular phase compared to the luteal phase [26,27,32]. 

Drug addiction brain dysfunction includes, among several brain structures, the nucleus accumbens (NAc) as important for engaging in initially rewarding behaviors and the dorsal striatum as associated with escalation and compulsive behaviors [37]. DA in these regions creates the craving or want to experience something new again, such as a new substance, and endorphins in the NAc serve to initiate the liking of the new experience. Therefore, when DA activation of the dorsal striatum is greater than the response seen in the NAc, pleasure decreases despite an increase in drug taking [38]. So, when intake progresses from a casual activity to a compulsive action, the pattern of activation in the brain also shifts from DA activation in the NAc to DA activation in the dorsolateral striatum [26]. In an attempt to resolve controversy regarding the causal contributions of mesolimbic dopamine (DA) systems to reward, there are three main competing explanatory categories: “liking”, “learning”, and “wanting” [39]. That is, DA may mediate (a) the hedonic impact of reward (liking), (b) learned predictions about rewarding effects (learning), or (c) the pursuit of rewards by attributing incentive salience to reward-related stimuli (wanting). Along these lines, Blum’s group evaluated these hypotheses, especially as they relate to the Reward Deficiency Syndrome (RDS), and they suggested that the incentive salience or “wanting” hypothesis of DA function is supported by most of the evidence. It is notable that File et al. examined the changes in substance- and behavior-related “wanting” and “liking” of human subjects, the key properties of Incentive Sensitization Theory (IST). Based on the Hungarian findings using structural equation modeling with 749 participants (503 women, *M_age_* = 35.7 years, *SD* = 11.84) who completed self-report questionnaires, “wanting” increased with the severity, frequency, and intensity of potentially problematic use, while “liking” did not change. Impulsivity positively predicted “wanting”, and “wanting” positively predicted problem uses/behaviors. Reward deficiency positively predicted problem uses/behaviors, and both impulsivity and problem uses/behaviors negatively predicted wellbeing [35]. Finally, women showed higher levels of “wanting” compared to men. This gender difference might be attributable to estrogen and other female-type hormones relative to male ones. Many factors interact to influence risk of drug dependence. As research in this field continues to evolve, a nuanced understanding of sex differences is critical for designing effective prevention and treatment strategies that account for the diverse needs of individuals.

### 3.2. Animal Studies 

Female rats have demonstrated greater drug-seeking behavior during abstinence, and escalate usage of cocaine, methamphetamine, and cannabinoids more easily than males [40]. They also showed greater drug intake and reinstatement [41]. Female rats during the estrus phase display more drug-seeking behavior and consumption than rats in the non-estrus phase [42] (Table 2). The estrogen-driven effects seen in drug addiction are evident in OVX rats displaying reduced acquisition, attenuated drug intake, and longer extinction than intact female rats [43,44]. These studies taken together show that ovarian hormones play important roles in the addictive behaviors of females and that they are more vulnerable than males to developing acquisition, maintenance, craving, extinction, and reinstatement. As shown by Table 2, OVX female rats had reduced conditioned place preference (CPP) behavior compared to intact females [45,46]. CPP tasks measure the motivational effects of a drug by comparing time spent in the drug chamber before and after conditioning. 

Exposure to prenatal stress may lead to a sex-dependent vulnerability towards drug addiction. Under basal conditions, the ability for females to acquire cocaine self-administration was higher than that of males [51]. The dorsal striatum, PFC, NAc, and medial extended amygdala had sexual dimorphisms. These regions contain gonadal hormone receptors that partly determine drug vulnerability on the basis of sex [52]. However, upon stress exposure, gonadal hormones can also determine sex-dependent vulnerability to drug addiction via differential activation of the HPA axis. Adult levels of gonadal hormones and developmental programming of the HPA axis can partially explain different stress responses between male and female subjects [53]. 

## 4. Effect of Estrogen on Drug Abuse

### 4.1. Ovarian Cycles

**Figure 1:**
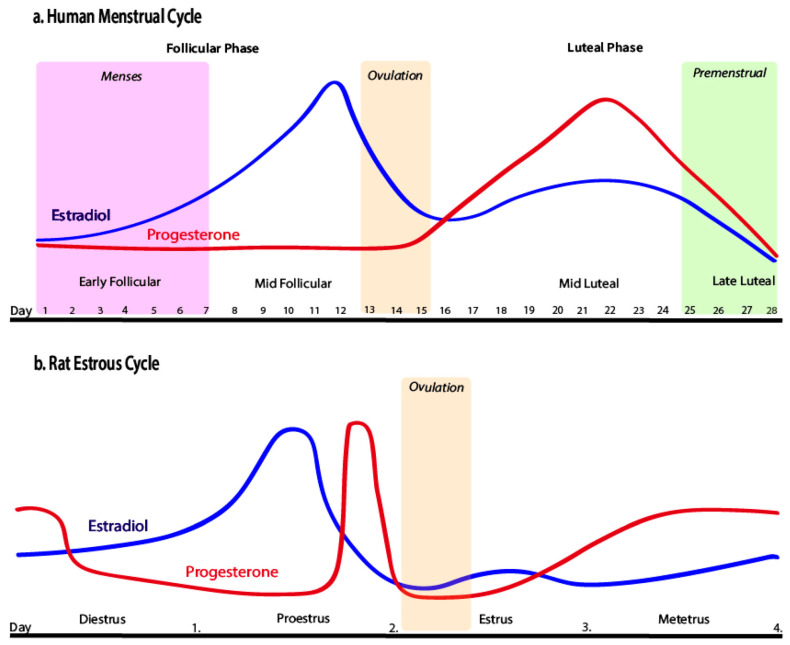
Human menstrual cycle and rat estrous cycle by Pestana et al. [55], licensed under CC BY 4.0. No changes were made.

Ovarian cycles play a key role regarding the vulnerability of female rodents to relapse/reinstatement, indicating the importance of ovarian hormones in determining susceptibility. Ovarian hormones interact with neural circuits activated during drug-primed, cue-instigated, and stress-induced relapse. Research on reinstatement has examined the rodent estrous cycle (see Figure 1b) and has shown that females in estrus typically have a greater reaction during cocaine-primed reinstatement when compared to their non-estrus counterparts [50,54,55]. Drug-primed reinstatement in females was greater in estrous compared to diestrus or proestrus females [47]. The opposite was seen when using drug-associated cues as priming agents; in this situation, estrus females showed a reduction in cocaine reinstatement [48,56]. These results were like the sex differences seen during reinstatement in which females had a greater response to drug-induced priming to cocaine and males had a greater response to cue-induced reinstatement. Gonadal hormone concentrations are greatly affected by the activity of DA. For the menstrual phase specifically (see Figure 1a), its effects on reward-associated neural capacity showed elevated activity during the midfollicular phase compared to the luteal phase, indicating that estrogen can direct mesolimbic function when uninterrupted by progesterone [55,57]. Despite their main role of supporting reproduction, these ovarian hormones evidently induce effects on other physiological and psychological systems, which can impact responses and adaptations to exercise. Most research studies have concentrated on hormonal fluctuations of the menstrual cycle during exercise, whereas many effects can be seen in an extended recovery period following exercise. 

### 4.2. Ovariectomy

A common effect of estradiol treatment on OVX rats is a greater reinstatement of responses for cocaine, which appears to be true for both acute and chronic estradiol administration [26]. OVX decreases cocaine intake when assessed in absence of estradiol. However, the effects of OVX on an escalation of cocaine use are restored by estradiol treatment [49]. Similar effects have been found concerning nicotine and alcohol self-administration under paralleled access conditions. Therefore, estradiol may be required for the development of an enhanced motivation for cocaine in females, as females without estradiol, such as those with OVX, did not exhibit this enhanced motivation for cocaine even while under conditions optimal for its development [28]. Another study found that OVX female rodents, when subjected to a forced swim test and tail suspension test, showed reduced immobility behaviors once treated with acute and chronic administrations of estradiol [58]. Comparatively, in humans, immobility would be associated as a possible symptom of depression, in which an individual may have trouble getting out of bed, refuses to engage in physical activity, and feels fatigued in general. It was determined that an abrupt withdrawal of ovarian hormones, as simulated by OVX, produces a quasi-depression state in females which can be reversed by estradiol administration, like an antidepressant [59]. To summarize, OVX female rats with no other treatment demonstrate a decrease in cocaine self-administration. But, when an OVX female is given estradiol, the rat will take more cocaine and work harder to obtain the cocaine, identically to an intact female during estrus [37].

### 4.3. Estrogen: Mechanisms of Action Males vs. Females

Differences in the DA systems of males and females are influenced by gonadal hormones. For females but not males, estrogen promotes DA release by altering the striatum and NAc. Estrogen receptors such as ERα and ERβ help establish sex differences in the brain and regulate TH mRNA formation, which controls the brain’s DA levels [18]. As previously mentioned, ovarian hormones modulate emotional behavior within the ECB system. Sex differences in the ECB system due to ovarian hormones therefore may explain those observed in symptoms of alcohol dependence [21]. For males, estradiol does not significantly affect cocaine taking, whereas testosterone levels are disrupted in response to drug use due to a decrease in DA levels. Many studies point to testosterone as being a key factor in the sex differences observed in patterns of drug abuse. Refer to the following review for information on the role of exercise on testosterone and implications for drug abuse [60]. Estradiol heightens acquisition of cocaine self-administration in OVX females, and those which are intact also acquire morphine and heroin self-administration and will work harder to obtain cocaine, morphine, and heroin than males [26]. Hormones associated with the menstrual or estrus cycle may enhance the initial reinforcing effects of drugs of abuse for females, thus contributing to a more rapid escalation of drug taking than for their male counterparts [37].

## 5. Treatments for Drug Abuse

### 5.1. Pharmacotherapy

A combination of clinical and preclinical studies has demonstrated the ability to use pharmacotherapy to target addiction-related systems [61]. Current pharmacological treatments specifically target receptors associated with the systems that drugs of abuse act on [62,63]. The goals of addiction pharmacotherapy are to normalize physiological processes that have been disturbed by long-term chronic drug use, minimize withdrawal symptoms, and lessen drug cravings which can result in relapse [64]. Physiological and cognitive withdrawal from psychoactive substances can be treated using pharmacotherapeutic agents. Currently, there are no FDA-approved pharmacotherapies for stimulant use disorder, including use of cocaine and amphetamines. Therefore, behavioral therapies are the main source of treatment, but use of antidepressants or antipsychotics may help to manage symptoms. Treatments for opioid withdrawal include μ-opioid receptor agonists and partial agonists such as methadone and buprenorphine that inhibit cAMP pathways, and α2 agonists like clonidine and lofexidine that reduce norepinephrine hyperactivity [65,66]. These are often useful treatments to manage withdrawal when dose reduction and discontinuation are difficult to achieve. Medical supervision is advised throughout this process [65]. Srivastava et al. (2020) further evaluates the clinical efficacy and utility of each of these options. Naltrexone is another medication used in the treatment of opioid use disorder, and it is also used to reduce cravings associated with alcohol use disorder by blocking endorphins, which can help to prevent relapse. On the other hand, Disulfiram works by creating an aversion to alcohol, which can help maintain abstinence [67]. Gupta et al. (2021) explores novel targets for alcohol withdrawal treatment [68]. SUDs influence various neurobiological pathways, and this heterogeneity is partially responsible for the multiple pharmacological options. Additionally, the concept that each treatment targets different stages of addiction, coupled with the understanding that individuals exhibit distinct responses to each intervention, further supports the availability of multiple pharmacological options to treat SUDs. 

While medications play an important role in the treatment of SUDs, preventative measures often focus on addressing the underlying causes and encouraging healthy behaviors and lifestyle choices, as well as reducing risk factors related to drug abuse and promoting mental wellbeing. Community and social-based initiatives, family support systems, education, and increased mental health resources strengthen protective factors and resilience to help reduce the risk of hazardous drug use [69,70,71]. Alternative non-pharmacological treatments such as cognitive behavioral therapy or exercise can also be used in conjunction with pharmacological treatments in treating substance abuse to enhance treatment and recovery using a holistic and personalized approach [70]. 

### 5.2. Exercise

Exercise is a non-pharmacological addiction treatment aimed at systems implicated in both early and late stages of addiction and has secondary health benefits. DA signaling is thought to be the primary mechanism motivating drug use [72], while glutamatergic signaling motivates drug use in later stages of the addiction process instead [73]. Physical activity is controlled by the mesolimbic DA pathway. Medial preoptic estrogen signaling changes DA response to the reward pathway by sending inhibitory signals to the VTA [64]. Pertaining to alcohol consumption, this may influence the status of estrogen via certain mechanisms, such as altered synthesis or metabolism of estrogen, causing levels in circulation to fluctuate, or changes in estrogen receptor signaling for target cells, which impacts how sensitive bone is to the hormone [74]. Exercise can be a risk factor in some cases because, if started later during drug abstinence, it can cause an increase in DA signaling, enhancing rather than attenuating drug seeking. The reward pathway additionally experiences an upregulation in glutamatergic signaling ensuing from repeated drug exposure and abstinence, which could cause a push towards drug seeking [75]. Much research though has found that exercise is a cost-effective and natural means to protect against vulnerability to drug use initiation, can enhance the brain’s resistance to addictive substances, and can prevent relapse. Exercise is beneficial at all stages of drug addiction and for a variety of substances, but more data are needed to directly examine the effects of timing of exercise initiation, exercise intensity, and exercise duration [75,76]. The main goal of using exercise as an auxiliary intervention is to improve affect, sleep, and self-esteem and reduce cravings and withdrawal symptoms. The research suggests this is best achieved by using low-intensity and high-frequency exercise to cultivate long-term habits as a substitute for drug dependence [69,77]. Adherence to exercise regimens is a challenge though so, often, contingency management and reinforcements are added to these treatments to enhance effectiveness of initiation and maintenance of exercise [78]. Voluntary wheel running, forced treadmill running, and forced swimming are the most common forms of exercise used in rodent drug addiction studies, and the efficacy of exercise may vary by sex [75]. Under similar levels of voluntary wheel running or forced treadmill running exercise, female mice have a larger adaptive capacity to increase their cardiac mass than males [79]. These results indicated that females are more likely to increase exercise capacity and hypertrophic response to exercise [79]. In terms of cocaine use, it has been demonstrated that short-term estrogen replacement attenuates cocaine-induced ambulatory activity, suggesting the involvement of estrogen membrane receptors [80].

Similarly, in forced swimming, there is greater activity seen in females; they swim at faster speeds and show fewer signs of fatigue. Yet it is ambiguous whether these sex differences correspond to gender-specific differences in response to stress, or whether females just prefer swimming as an exercise [79]. Several studies have also revealed that exercise has the capability to reduce symptoms of drug addiction by reducing cognitive decline, which is caused by the production of new neurons in the hippocampus. However, these findings were observed to be more prominent in females than in males [18].

## 6. Exercise and Estrogen

### 6.1. Wheel Running

**Figure 2:**
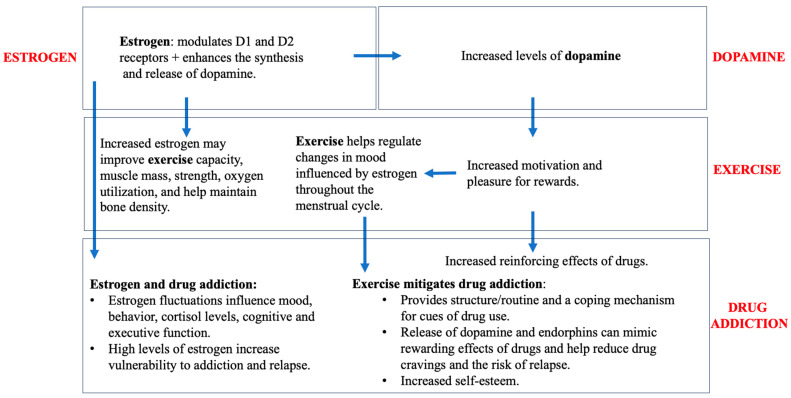
Impact of exercise, estrogen, and dopamine on drug addiction.

Studies have shown that there are exercise-induced alterations in the DA system that mediate the rewarding effects of drugs, resulting in reduced drug-seeking behaviors and negative health outcomes and enhanced cognitive function. Lower levels of D1R and higher levels of D2R reportedly help to mitigate drug-seeking behaviors, making exercise a useful treatment for substance abuse [76]. Figure 2 provides a schematic of the relationship between estrogen, dopamine, and exercise and drug addiction, starting with estrogen’s effect on dopamine, which impacts exercise and drug addiction. Estrogen’s effects on exercise and drug addiction are also briefly summarized. Estrogens are believed to enhance the voluntary wheel running of rodents via medial preoptic (mPOA) ERα signaling. Injections of an ERα agonist increased wheel running in OVX rats, while those of the ERβ agonist had no effect [81,82,83]. The behavior outcomes of ER-mediated cell signaling is believed to be a cyclic process. Thus, if mPOA ERβ regulates ERα signaling, it is most likely not specific to wheel-running behavior [84]. Such results are summarized in Table 3. In another study, concurrent access to a running wheel decreased initiation of alcohol consumption under two-bottle free-access conditions, which alludes to the point that exercise may serve in competition with the drug to help reduce vulnerability as an alternative non-drug reinforcement [85]. The two-bottle choice paradigm is widely used in preclinical studies to evaluate the effect of a substance on an animal’s voluntary consumption, which is important when evaluating the rewarding properties of a drug. Animals are provided with access to two bottles, one containing the drug being investigated. Researchers can track changes in animal drinking behavior and preferences by examining various concentrations. While under the 24-h free-choice condition, animals were seen to have decreased consumption after wheel running, but not under models in the dark, as an increased level of consumption was induced. These data suggest that exercise can decrease positive reinforcing effects of various drugs, with efficacy varying according to exposure and the individual [85]. Moving on to cocaine, animals which underwent chronic voluntary exercise administered less cocaine during cocaine self-administration; they also showed a lower escalation of cocaine intake compared to sedentary rats [86]. This effect was significant for females, not males. It has been suggested that females require a greater volume of exercise to see equal reductions in drug-seeking behavior compared to their male counterparts [76]. In regards to alcohol, voluntary wheel running decreased consumption, but consumption returned back to baseline levels under forced wheel running [87]. During abstinence, 24-h access to the running wheel failed to block the typical enhanced alcohol consumption afterwards, as it instead favored higher consumption in the wheel-running group when compared to those of the sedentary control group [75]. 

### 6.2. Treadmill

In one study, female mice were found to have a greater capacity for exercise due to estrogen and nitric oxide under a treadmill regimen. Male mice ran significantly less than female mice, and female mice contributed 20% more work to exhaustion [50]. However, when the animals were age-matched, the results showed that males had greater weight and skeletal muscle mass, impeding their running. A weight-matched comparison was also performed to analyze the exercise capacity of male and female mice [50]. Even at varying ages, body-weight-matched female mice still ran farther and had a higher work to exhaustion. Further matching was performed for skeletal muscle mass. These results agreed with the other analyses; females ran 11% farther and performed 14% more work to exhaustion [50]. Meanwhile, if the females were OVX mice, this enhanced exercise capacity was lost as they ran similar distances to intact males. Furthermore, when males were treated with estrogen, their exercise capacity resembled that of intact females [50]. Nitric oxide synthase, a later target of estrogen, helps enhance exercise capacity in intact females and males treated with estrogen. Female mice originally had greater nitric oxide synthase activity than males, but once they had undergone OVX, their activity became very similar to that of the males [88]. Males treated with estrogen were found to have a similar activity of nitric oxide synthase as intact females. When nitric oxide was blocked, these sex differences in exercise capacity were no longer seen. OVX causes the decline in estrogen levels in the cerebellum; this decline can be prevented with exercise [88]. Cerebellar estrogen levels have also shown to play a role in motor coordination performance. Either way, the mechanism between exercise and cerebellar estrogen is not fully known and needs further investigation [88]. However, regular exercise has been proven to improve the spatial memory of OVX rats as decreases in estrogen levels are known to lead to dysfunction of the hippocampus. In one such case, the spatial memory retention and hippocampal estrogen level of the OVX exercise group were significantly better than in the OVX control group [89]. In a recent clinical study, eumenorrheic, trained female runners underwent a treadmill regimen to measure the effect of the menstrual cycle on running economy. It was determined that the mid-luteal phase caused increased core temperature and minute ventilation, and that running economy in this phase is significantly impaired at intensities applicable to performance training [90]. Therefore, if this is applied to women undergoing withdrawal from drugs of abuse, it would most likely be advisable to begin exercise training after the mid-luteal phase. 

## 7. Limitations and Future Directions 

More attention should be drawn to exercise intervention for drug addiction and how its effects depend on the sex of the individual. Ideally, future preclinical studies involving drugs of abuse should combine exercise treatment with ovariectomy and subsequent estrogen treatment to gather more direct data on this topic. More studies involving the combined measurements of estrogen, exercise, and drug abuse need to be conducted. For example, one possible study could involve both an exercise and sedentary group of female rats, all undergoing CPP for cocaine. Each of those groups could be further divided into OVX and non-OVX groups, with the OVX groups additionally being readministered with a set dose of estradiol later. The tendency to ignore sex differences may contribute to the limitations of developing more effective exercise treatments for drug abuse. Since male animals have been used almost exclusively in preclinical studies, biases arise which can lead to unforeseen outcomes when applied to a clinical trial including female participants. While conducting this review, we acknowledge that there is a wide range of pertinent literature and additional studies that could have improved the comprehensiveness of our analysis. However, to adhere to the journal’s guidelines, we had to limit the number of studies included in this review. Additionally, while this review contains valuable information regarding the effects of sex and exercise on drug addiction, we acknowledge that a more exhaustive synthesis of the evidence is needed to provide a fully comprehensive and objective understanding of the research utilizing statistical analysis of the methods and results of included studies. The heterogeneity of study designs and outcome measures may impact the generalizability of such findings and implications.

## 8. Conclusions

Exercise alone has several benefits and can mitigate the risk of many diseases. It has been found that exercise-induced alterations in the mesolimbic dopamine pathway, specifically when there is decreased D1R-like binding and/or increased D2R-like binding, play a role in the attenuation of drug-seeking behaviors, thereby making exercise an effective addition to the prevention and treatment of substance abuse and relapse. As previously stated, much research, both preclinical and clinical, has been carried out on male subjects, yet it has been shown that there are sex differences in the effects of exercise on dopaminergic responses and, therefore, efficacy against substance use. This narrative review analyzes the role of estrogen in this association while providing an overview of general mechanisms of substances and their relation to estrogen to further support the role of sex in drug addiction. It has been shown that estrogen enhances the rewarding effects of drugs in females, contributing to a more rapid escalation of drug taking than in males and more difficulty with drug abstinence, resulting in greater risk of relapse.

Pertinent pharmacological treatments are reviewed, and non-pharmacological treatments such as exercise paradigms, including treadmill and wheel running, have been shown to attenuate drug-seeking behaviors. In female rats specifically, effects of the estrous phase are observed on mesolimbic function, which has implications for drug addiction. Response and efficacy to exercise paradigms were observed, and these studies were modeled after clinical regimens; therefore, it has been found that females often require exercise at greater volumes compared to males to obtain similar results, and forced paradigms that induce stress may be included to replicate patterns seen in humans. Utilizing low-intensity and high-frequency aerobic exercise with other treatments has been emphasized to aid in the long-term efficacy of treatments by establishing a safe and healthy routine in place of drug dependence that can provide a supportive environment that aids in self-esteem, positive affect, and executive function while reducing stress, drug cravings, and relapse. The role of estrogen in dopaminergic regulation, exercise, and drug addiction pathways is multifaceted and complex. As such, a single and definitive conclusion is challenging to deduce because of the nuanced and dynamic nature of these relationships. Because of the sex differences observed in exercise-induced DA modulations, sex-specific treatments involving comprehensive investigations must be developed to aid in the treatment of substance abuse and to better elucidate these connections. 

## Figures and Tables

**Table 1:** Effects of menstrual cycle phase on drug response.

Drug	Menstrual Phase	Impact on Drug Abuse: Effects on Behavior and Mood	References
Cocaine	Follicular	↑ mood altering effects: wanting more, energy feelings of euphoria.	[32,35]
	Luteal	↑ symptoms of dysphoria	[32,36]
Alcohol	Follicular	↑ anxiety and depression ↑ consumption overall ↑ consumption in those with premenstrual syndrome compared to control. ↑ consumption in those on hormonal contraceptives compared to control	[32,33]
	Luteal	↑ anxiety ↑ consumption	[32]
	No cycle differences. No significant effect of cycle phase on consumption/relationship inconclusive		[32] [33]
Opioids	Follicular	↑ sensitivity to adverse effects	[34]
	Luteal	↑ cortisol and prolactin responses Significantly ↑ sensitivity to adverse effects compared to FP and men	[34]
	No cycle differences		[32]
Nicotine	Follicular	↑ depressive symptoms, relapse tendency, physiological reactivity	[26,27]
	Luteal	↑ stimulation and cognitive task performance ↓ urge to smoke and reactivity	[27,32]

**Table 2:** Estrogen levels of female rats influence cocaine administration.

Estrogen Level	Strain/Age	Cocaine Dose	Cocaine Self-Administration	References
Intact during estrus phase (high levels of estradiol)	Sprague-Dawley rats	0.5 mg/kg per infusion	Increases	[47]
		0.5 mg/kg per infusion	Increases	[48]
Intact during non-estrus phases (low levels of estradiol)	Sprague-Dawley rats	0.5 mg/kg per infusion	Decreases	[47]
		0.5 mg/kg per infusion	Decreases	[48]
Ovariectomized	Sprague-Dawley rats about 3 months of age	1.5 mg/kg per infusion	Decreases	[49]
		0.4 mg/kg per infusion	Decreases	[50]
Ovariectomized with estradiol treatment	Sprague-Dawley rats about 3 months of age	1.5 mg/kg per infusion	Increases	[49]
		0.4 mg/kg per infusion	Increases	[50]

**Table 3:** Estrogen and its effects on voluntary wheel running in female rats.

Estrogen Receptors	Voluntary Wheel Running	References
ERα signaling in non-OVX	Increases	[81,82,83,84]
ERβ signaling in non-OVX	Decreases slightly	[81,84]
ERα agonist in OVX	Increases	[81]
ERβ agonist in OVX	No effect	[81]
ERα and ERβ agonists in OVX	No effect	[84]

## Data Availability

All data is available on request.
